# Cryptobenthic reef fishes may not be safe from invasive lionfish (*Pterois* spp. Oken, 1817)

**DOI:** 10.1111/jfb.70636

**Published:** 2026-09-01

**Authors:** Liana F. Mendes, Ariston P. Santos, Thaisa A. Souza, Lorena S. Agostinho, Ana B. A. Bennemann, Jorge Cortés, Maria S. Toro, Juliana S. S. Luz, Clara B. P. E. Silva, Carlos. E. R. D. Alencar, Priscila F. M. Lopes

**Affiliations:** ^1^ Departamento de Ecologia (DECOL‐CB) Universidade Federal do Rio Grande do Norte (UFRN) Natal Brazil; ^2^ Programa de Pós‐Graduação em Ecologia (PPG‐ECO), Centro de Biociências (CB‐UFRN) Universidade Federal do Rio Grande do Norte (UFRN) Natal Brazil; ^3^ Centro de Investigación en Ciencias del Mar y Limnología (CIMAR) Universidad de Costa Rica San José Costa Rica; ^4^ Centro Comunitario de Buceo Embajadores del Mar Puerto Viejo Costa Rica; ^5^ Departamento de Ciências Biológicas (DCBio) Universidade Regional do Cariri (URCA) Crato Brazil; ^6^ Research Institute of the University of Bucharest (ICUB) University of Bucharest Bucharest Romania; ^7^ Institute of Biological Research Cluj National Institute of Research and Development for Biological Sciences Cluj‐Napoca Romania

**Keywords:** benthic habitat, Blenioidei, Brazil coast, Costa Rica Caribbean, Gobioidei, marine protected area

## Abstract

Lionfish (*Pterois* spp.) are hypothesized to negatively influence cryptobenthic reef fish assemblages through predation. We evaluated these potential effects on Caribbean reefs in Costa Rica and tropical reefs in Brazil. Brazil provided a reference condition for cryptobenthic assemblages prior to lionfish invasion, while the Costa Rica sites enabled comparisons between reefs inside marine protected areas (MPAs), where lionfish removal did not occur for several years, and adjacent fished reefs where removals occurred. Visual censuses (30 m × 2 m transects, 0.5–4 m depth) were complemented by simplified assessments of reef structure and benthic cover. Cryptobenthic fish abundance was consistently higher in Brazil than in Costa Rica. Within Costa Rica, abundance was significantly lower inside MPAs, where higher lionfish densities were recorded, compared to adjacent fished reefs. This pattern indicates differences in cryptobenthic fish abundance across sites with contrasting lionfish presence. Our findings reinforce concerns that MPAs may inadvertently function as refuges for invasive species, highlighting the importance of adaptive management strategies, including targeted removals and monitoring, to mitigate ecological impacts and protect reef biodiversity.

## 1 INTRODUCTION

The lionfish (*Pterois* spp. Oken, 1817) is a voracious invasive fish species that can have a significant impact on ecosystems, particularly by disrupting local food chains (Chagaris et al., 2020), when found outside its natural habitat in the Indo‐Pacific region (Albins & Hixon, 2013). Its invasive behaviour was first documented in Florida, USA, during the 1980s, and it is believed to have spread throughout the Caribbean Sea from there (Morris Jr, 2013). The fast trajectory of this invasion reveals its impressive adaptive capacity attributed to several natural history traits, including its high reproductive potential, which allows it to produce a large number of eggs and larvae, efficient anti‐predation defences due to its venomous spines and aposematic coloration (Morris Jr, 2013), and its generalist feeding behaviour (Batjakas et al., 2023). Lionfish are opportunistic predators, feeding on a wide variety of fish and invertebrates (Cure et al., 2014; Green et al., 2012; Morris Jr, 2013; Peake et al., 2018). Even small cryptobenthic fish are not immune to the lionfish's voracious appetite; for instance, in the Caribbean region of Costa Rica, cryptobenthic fish rank as the third most consumed prey by lionfish (Sandel et al., 2015). This predatory pressure on key species raises concerns about the balance of reef communities and the resilience of ecosystems.

Cryptobenthic fishes are a diverse and species‐rich group, comprising 17 families and 2799 identified species. Approximately 44% of species are smaller than 10 cm and exhibit cryptic coloration and behaviour. They are closely associated with the benthic substrate, inhabiting holes and fissures that provide shelter within complex reef structures (Brandl et al., 2018; Depczynski & Bellwood, 2005). Owing to their diversity and life‐history traits, this group also shows relatively high levels of endemism (Brandl et al., 2018), including in Brazil, where 97 species have been documented, approximately 50% of which are endemic (Pinheiro et al., 2018), with several species having been studied and described in the last decades in Brazil (e.g., Carvalho‐Filho et al., 2020; Dalben & Floeter, 2012; Mendes, 2006; Mendes, 2007; Neves et al., 2016; Rangel & Mendes, 2009; Sampaio et al., 2004). Despite their low biomass, cryptobenthic fishes contribute substantially to reef productivity due to high metabolic rates, rapid growth, short life cycles, high fecundity and dynamic larval dispersal (Brandl et al., 2018, 2019). They play an important role in reef ecosystem functioning by linking primary production to higher trophic levels through the consumption and recycling of organic material (Ahmadia et al., 2012; Dalben & Floeter, 2012; Depczynski & Bellwood, 2004). However, this group remains underrepresented in reef fish research, partly due to their small size and cryptic microhabitat use, which complicate observation and sampling (Beldade & Gonçalves, 2007; Brandl et al., 2018, 2019; Dalben & Floeter, 2012; La Mesa et al., 2006; Neves et al., 2016).

These fishes are often restricted to specific microhabitats characterized by substrate rugosity, habitat complexity and the availability of small crevices and refuges (Dalben & Floeter, 2012; Depczynski & Bellwood, 2005; Patzner, 1999), which provide protection against predators (Harborne et al., 2012; Horn et al., 1999). However, in the Caribbean, such refuges may not fully protect them from invasive lionfish predation (Sandel et al., 2015), which appears to bypass previously effective defensive strategies, with potential ecological consequences that remain poorly understood. This highlights the importance of improving baseline knowledge and implementing early management actions to mitigate impacts on reef ecosystems.

Since 2020, regular observations of lionfish have been reported in the warm waters of South America (Luiz et al., 2021), with new sightings and captures in oceanic islands (Fernando de Noronha Archipelago and Atol das Rocas) and coastal areas of northeastern Brazil (Maggioni et al., 2023). There is currently no official action plan or protocol for controlling the species at a national level in this region. This lack of guidance hampers local response efforts, leading to gaps in the proper handling and disposal of captured individuals. Most lionfish captures in the region are carried out by artisanal fishers, typically using freediving or compressor diving techniques (pers. obs.), but these fishers often lack technical support on how to deal with the species. The situation is further compounded by the absence of sufficient Marine Protected Areas (MPAs), which significantly limits the implementation of formal control strategies.

On the other hand, MPAs, established to conserve and restore marine biodiversity, may inadvertently create favourable conditions for invasive lionfish. By providing abundant prey and restricting human activity, MPAs can facilitate lionfish colonization, growth and reproduction (Kleitou et al., 2024). In the absence of continuous removal efforts, lionfish densities inside MPAs may surpass those of surrounding unprotected areas, leading to sharper biodiversity declines within protected zones (Soares et al., 2025). Moreover, MPAs can potentially act as source areas, exporting lionfish eggs, juveniles and adults to neighbouring ecosystems (Crec'hriou et al., 2010).

Evidence shows that targeted removal reduces lionfish density by about 60%, whereas areas without removal have shown increases of 200%–400% (Kleitou et al., 2024). These findings reinforce the urgent need for legislation permitting lionfish control within MPAs (Soares et al., 2023; Soares et al., 2025). Such measures do not undermine the purpose of MPAs but highlight the need for adaptive management, including strategic, periodic removals to suppress invasive populations. Yet, most Southwestern Atlantic MPAs (88%) currently lack regular culling programs, inadvertently fostering lionfish persistence and spread (Soares et al., 2025).

This study evaluates the composition and abundance of cryptobenthic fish communities in relation to lionfish presence in two regions of the Western Atlantic: Costa Rica, where lionfish have been established for over 20 years (Sandel et al., 2015), and the Brazilian coast, which is experiencing a recent invasion, with the species reported in ~60% of the coastline and two oceanic islands over the past 5 years (Maggioni et al., 2023). At the time of our surveys, lionfish were absent from the Brazilian sites, providing a reference condition for cryptobenthic assemblages in reefs not yet exposed to the invader. In contrast, Costa Rica offered the opportunity to assess long‐term invasion impacts, as well as differences between management regimes. There, we compared reefs inside an MPA, where lionfish removal did not happen during the study period, with adjacent unprotected areas where artisanal fishing and recreational diving frequently capture lionfish specimens, which possibly helps to control the populations. These contrasting scenarios allowed us to evaluate both the early‐ and late‐stage consequences of lionfish invasion, while also testing how management interventions, or their absence, shape cryptobenthic fish communities.

## 2 MATERIALS AND METHODS

### 2.1 Study area

All data used in this research were collected through visual censuses conducted during diving expeditions on tropical reefs along the Brazilian equatorial coast (particularly Rio Grande do Norte state) and in the Caribbean region of Costa Rica (from Manzanillo to Isla Uvita) (Figure 1). The same standardized methodology was applied in both countries, with data collection conducted at depths ranging from 0.5 to 4 meters (see below). Visual censuses were conducted in Brazil before the arrival of the lionfish (2015 and 2016), and the research in Costa Rica was conducted in 2017 and 2018.

**FIGURE 1:**
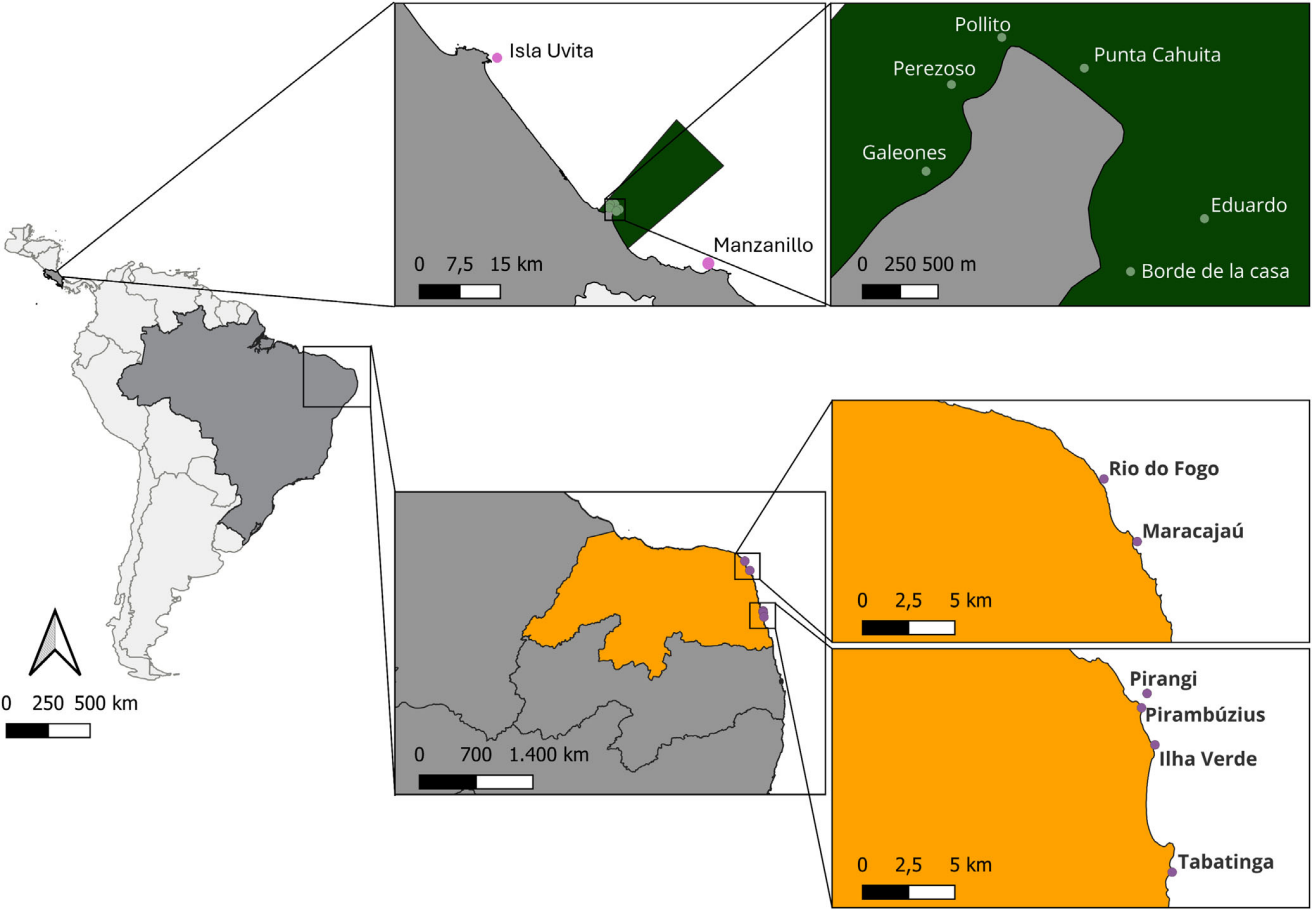
Study sites in Brazil and the Costa Rican Caribbean. In Costa Rica, the sites located within Cahuita National Park (green polygon) are marked with green points, while the Isla Uvita and Manzanillo sites are marked with pink points. In Rio Grande do Norte, Brazil (orange), the collection sites of Rio do Fogo, Maracajaú, Pirangi, Pirambúzios, Ilha Verde and Tabatinga are marked with purple points.

The Caribbean region of Costa Rica is characterized by coral cover in its reef zones, which can be classified into three main areas: (1) fringing and patch reefs located between Moín and Limón; (2) fringing reefs, patch reefs and carbonate banks within Cahuita National Park (CNP); and (3) fringing reefs, patch reefs, carbonate banks and algal ridges between Puerto Viejo and Punta Mona (Cortés, 2016; Cortés et al., 2010). A recent decline in live coral and coralline algae cover has been observed, while non‐coralline and turf algae have increased, dominating the substrate (Cortés et al., 2010). In this study, we focused on two distinct areas in the Costa Rica Caribbean region: (1) inside Cahuita National Park (CNP), a marine protected area (MPA) known for its well‐developed coral reefs, and (2) outside the boundaries of CNP. Inside the CNP, due to regulations governing access and usage, efforts to remove lionfish have not been consistent. Conversely, outside the park, fishing and SCUBA diving tourism activities are linked to frequent lionfish catches.

The tropical reefs along the coast of Rio Grande do Norte, Brazil, are known for their diverse composition, often characterized by the dominance of macroalgae (Feitosa & Ferreira, 2015; Zilberg et al., 2016). In some areas, sponges are significantly represented (Figueiredo et al., 2008; Mendes & Grimaldi, 2020), while other sites have greater coral cover, such as the Maracajaú reefs and Rio do Fogo (Laborel‐Deguen et al., 2019). At the time of data collection (2015–2016), there were no recorded instances of lionfish on the Brazilian coast. Therefore, these sites were used as a reference condition representing reef fish assemblages in the absence of lionfish, while acknowledging the biogeographic and ecological differences between regions. As such, comparisons with Caribbean reefs should be interpreted cautiously, as differences in community structure may also reflect underlying environmental and ecological variation. Nevertheless, tropical reef fish assemblages across the Atlantic share comparable functional guilds (Floeter et al., 2008), allowing for broad, albeit limited, comparisons.

### 2.2 Assessing cryptobenthic fish species and their substrate

Cryptobenthic fish abundance was assessed by counting individual per species across three study conditions: (1) the Brazilian coast (reference condition, no lionfish records at the time of sampling), (2) reefs outside the Marine Protected Area (off CNP) and (3) reefs within the MPA (CNP). Surveys were conducted using 30 m × 2 m belt transects. A total of 130 transects were surveyed, comprising 60 transects (3600 m^2^) along the Brazilian coast and 70 transects (4200 m^2^) in Costa Rica, of which 50 were located within the MPA and 20 outside. Transects were positioned randomly within each site. This methodology was adapted from evaluation protocols used for assessing reef fishes (e.g., www.agrra.org and www.reefcheck.org), decreasing the width for more accurate recording of these small fish species. Fishes were surveyed and identified in situ by an experienced observer trained in cryptobenthic fish identification (e.g., Mendes, 2006; Mendes, 2007; Rangel & Mendes, 2009). Surveys involved active searching of microhabitats, including burrows, crevices and spaces within algal cover, to improve detection of cryptic species. Species identification was based on visual characteristics following established taxonomic references for reef fishes. Although transects were designed to target reef fishes, the 30 m × 2 m dimensions are appropriate for recording conspicuous species such as lionfish, which were readily detected within the surveyed area.

To account for potential habitat‐related influences on cryptobenthic fish assemblages between Brazil and Costa Rica, reef architecture (HAS—Habitat Assessment Score; Gratwicke & Speight, 2005) and benthic cover (PIT—Point Intercept Transect; Reef Check Protocol, 1996) were quantified across all sites. This is particularly relevant given the strong association between cryptobenthic fishes and substrate characteristics (Depczynski & Bellwood, 2003). These measurements allow for a more consistent comparison among sites by accounting for variation in habitat structure that may influence assemblage composition independently of lionfish presence. Other environmental and ecological factors known to influence cryptobenthic fish assemblages, such as seasonality, hydrodynamics, temperature and the composition of other fish communities (e.g., Brandl et al., 2018; Depczynski & Bellwood, 2003; Horn et al., 1999), were not explicitly quantified in this study, but should be considered when interpreting the observed patterns.

Benthic cover was assessed using the PIT method, adapted from the Reef Check Protocol (1996). Points were recorded at fixed intervals along each transect, and each point was assigned to a predefined benthic category (e.g., hard coral, macroalgae, sponge or other substrate types). This approach allows for the estimation of percent cover and spatial variation in benthic composition (Hill & Wilkinson, 2004). Points were recorded along each transect, with a total of 100 points evenly spaced at 20 cm intervals along the 20 m transect. Each point was assigned to a specific category representing a different type of bottom cover. By counting the number of points in each category, the percentage of bottom cover for each category was determined. The categories considered included: foliose algae (foa), calcareous algae (caa), filamentous algae (turf), articulated algae (aa), sponges (spo), hard corals (hc), zoanthids (zoan), sand (san), gravel (gra), rock (roc), dead coral (deco), diseased coral (dico), octocorals (oct) and seagrass (seag).

Reef architecture was evaluated using the HAS method (Gratwicke & Speight, 2005), based on the visual estimation of structural attributes relevant to habitat complexity. The following variables were assessed: rugosity (RUGOSI), plant and animal growth form variety (FORMS), height (HEIGHT), refuge categories (REFUGE), living cover (LCOVER) and hard substrate (HSUBST), each scored on an ordinal scale. Data were collected from five 1 m^2^ quadrats positioned along each transect (total = 650 quadrats), and mean values per transect were subsequently calculated.

### 2.3 Statistical analyses

Before conducting the statistical analyses to assess differences in substrate and bottom cover, the abiotic data obtained from the HAS and PIT environmental matrices were evaluated for the presence of outliers, following the guideline outlined in Zuur et al. (2010). Outliers in the abiotic data were identified using a Cleveland plot (Cleveland, 1993) and principal component analysis (PCA), as recommended by Kohler and Luniak (2005). The abiotic data were then standardized using the z‐score method, which transforms the data to have a mean of 0 and a variance of 1 (Gotelli & Ellison, 2011).

The PCA was performed separately for the HAS and PIT datasets to examine environmental variation among the reefs in Brazil and Costa Rica (inside and outside the CNP), focusing on the main components that explained a cumulative variance of 70% (Lima et al., 2014; Pereira et al., 2017). The load values (loadings) of the variables were also analysed, with modular values above 0.4 considered indicative of relevant variation and included in the discussion of abiotic data (Rakocinski et al., 1996). After assessing the overall variation in the data, a discriminant function analysis (DFA) was conducted using a stepwise forward algorithm (Timm, 2002) separately for HAS and PIT, with the location classifiers (Brazil, Costa Rica CNP, Costa Rica off CNP) to identify the abiotic variables that best discriminated between the groups.

To assess the composition of cryptobenthic fish (richness and abundance), we used several biodiversity descriptors, including dominance, Simpson's diversity index, Shannon's diversity index and Pielou's evenness (Magurran, 2004). We performed bootstrap resampling with 999 iterations to obtain 95% confidence intervals and compared the diversity descriptors among the different study areas (Brazil vs. Costa Rica inside and outside the CNP) using Monte Carlo permutation tests (*n* = 9999) (Manly, 2018). The Odds Ratio test was applied to determine the association between the presence of lionfish and changes in the fish community. Additionally, we employed an Analysis of Similarities (ANOSIM), based on the Jaccard index, to assess compositional dissimilarities among the study areas (Anderson, 2008), using presence/absence data for cryptobenthic fish species together with lionfish records. This analysis evaluated spatial variation in fish assemblage composition across the study areas. All analyses were done in R (R Core Team, 2021).

### 2.4 Ethics statement

The research did not involve any animal capture, handling or experimental manipulation. Observations were conducted using non‐invasive visual census and point‐intercept transect techniques in accordance with local and national regulations. As no animals were manipulated, ethical approval was not required.

## 3 RESULTS

### 3.1 Abundance of cryptobenthic fish in tropical Atlantic reefs

The abundance of cryptobenthic fish was consistently higher in Brazil than in Costa Rica, both inside and outside of the CNP (CNP Bootstrap, *n* = 999; *p* < 0.01; Off CNP Bootstrap, *n* = 999; *p* < 0.01) (Figure 2a,b). Yet, within Costa Rica, the abundance of cryptobenthic fish was higher outside the protected area compared to areas inside MPA (Annex S1, Figure 2b). Regardless of protection status, sites with lionfish presence consistently exhibited lower cryptobenthic fish abundance (Odds ratio = 0.0625; z = −23.468; 95% CI = 0.006169–0.6332; *p* = 0.0189; Bootstrap, *n* = 999; *p* < 0.01). However, there was no significant difference in cryptobenthic fish richness between sites with and without lionfish (Bootstrap, *n* = 999; *p* = 0.7689). Biodiversity descriptors, including dominance, Simpson's diversity index, Shannon's diversity index and Pielou's evenness, did not differ significantly among areas, with the exception of Pielou's evenness between Brazil and sites outside the MPA (Brazil: 0.9807; outside CNP: 0.8180; bootstrap, *n* = 999; *p* < 0.01). The ANOSIM analysis, based on the presence/absence of cryptobenthic fish species together with lionfish records, indicated differences in assemblage composition between Brazil and the Costa Rican sites, whereas no significant difference was detected between sites inside and outside the MPA (ANOSIM, *p* = 0.0954) (see Supplementary Material, Annex S2 and S3).

**FIGURE 2:**
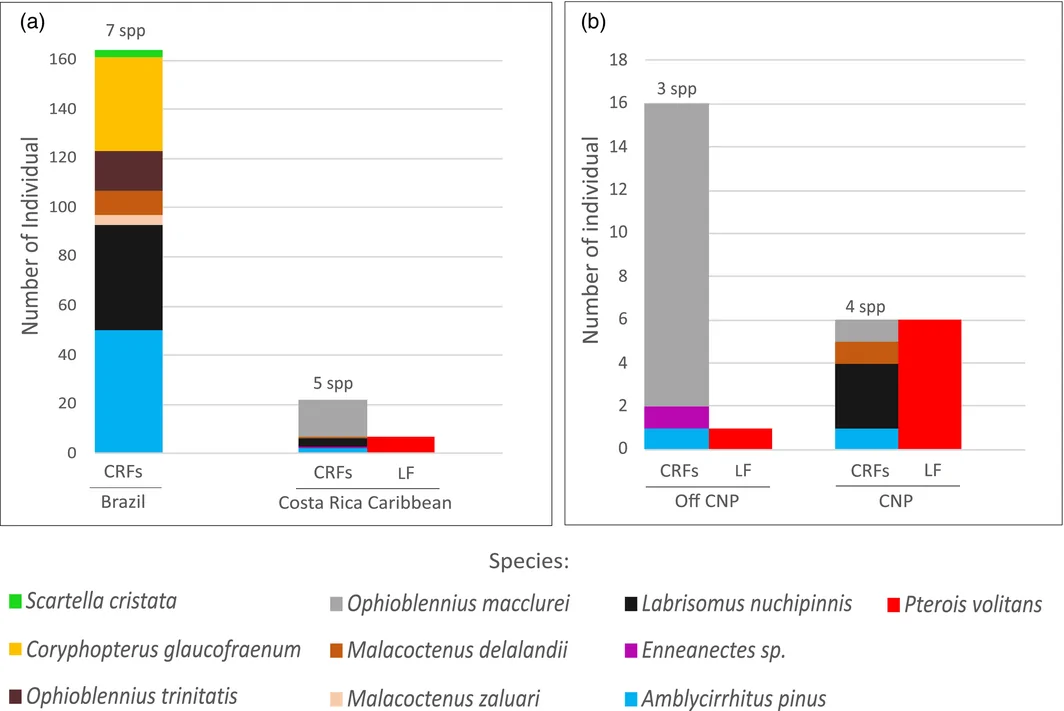
(a) Abundance of cryptobenthic reef fishes (CRFs) and lionfish (LF—*Pterois volitans*) in the tropical reef areas of Brazil (3600 m^2^) and the Caribbean of Costa Rica (4200 m^2^). The bars represent the observed abundance, with species richness indicated above the bars. (b) Abundance of CRFs and lionfish in Costa Rica only, comparing areas inside (3000 m^2^) and outside (1200 m^2^) the Cahuita National Park (CNP).

In Brazil, seven species were recorded, with a total of 164 individuals. The most abundant species were *Amblycirrhitus pinos* (*n* = 50), *Labrisomus nuchipinnis* (*n* = 43) and *Coryphopterus glaucofraenum* (*n* = 38). In Costa Rica, outside the protected area, species richness was lower (4 spp.), with *Ophioblennius macclurei* as the most abundant (*n* = 14). One lionfish individual (*Pterois volitans*, *n* = 1) was also recorded. Within the protected area, five species were recorded, but overall abundance was low. Lionfish were the most abundant species (*n* = 6), while among native species only *L. nuchipinnis* had more than one individual (*n* = 3) (Figure 2a,b).

In the reef architecture analysis (HAS) (Figure 3a), the first two main components added up to 73.4% (PC1 = 55.1%; PC2 = 18.4%) and indicated that the reef areas tend to show different profiles regarding these attributes. According to the DFA analysis, the presence of hard substrate (HSUBST), living cover (LCOVER) and substrate height (HEIGHT) were the variables that, in descending order, showed discriminatory power for distinguishing reef profiles. Although RUGOSI, FORMS and REFUGE contributed to the multivariate variation captured by the PCA (Annex S4), they were not retained by the DFA as the variables with the greatest discriminatory power among regions. Their values were consistently high across all study areas, suggesting that each region contained structurally complex reefs despite differences in other habitat attributes, particularly hard substrate, living cover and substrate height.

**FIGURE 3:**
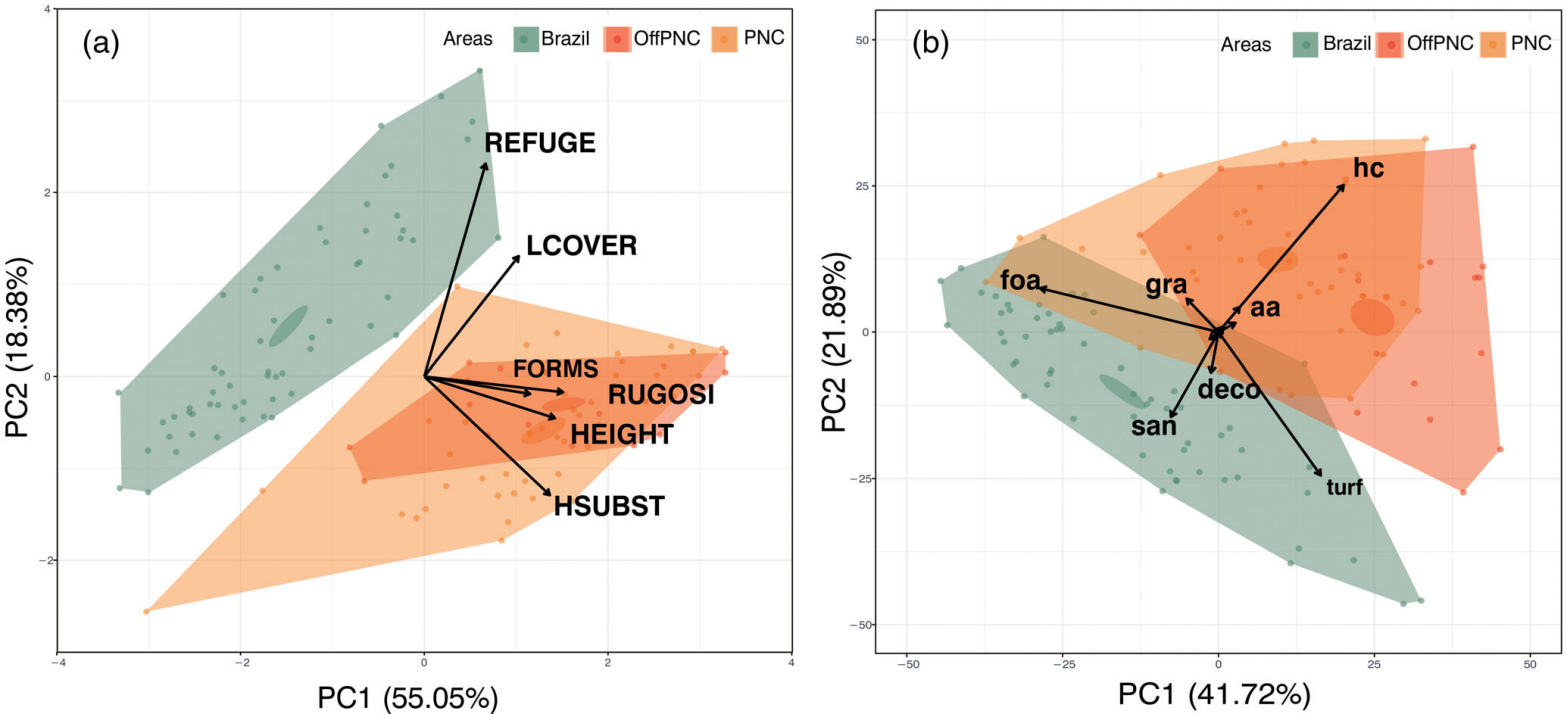
(a) Principal component analysis for reef architecture (HAS) data with visualization of groups by areas (Brazil and Costa Rica Caribbean, inside and outside Cauhita National Park—CNP). Polygons join the most extreme points of variation by groups. Ellipses indicate the centre of dispersion for each of the groups. Rugosity (RUGOSI), plant and animal growth form variety (FORMS), height (HEIGHT), refuge categories (REFUGE), living cover (LCOVER) and hard substrate (HSUBST). (b) Principal component analysis for Point Intercept Transect data with visualization of groups by location (Brazil—green, Costa Rica—warm colours). Polygons join the most extreme points of variation by groups. Ellipses indicate the centre of dispersion of each group. Foliose algae (foa), articulated algae (aa), hard corals (hc), dead coral (deco), gravel (gra), filamentous algae (turf), sand (san).

In the evaluation of bottom cover attributes (PIT classification) (Figure 3b), Brazil and Costa Rica exhibit a wide diversity of substrates with a trend towards distinct profiles (distant centroids). The first three principal components accounted for 81% of the data (PC1 = 41.7%, PC2 = 21.89%, PC3 = 17.2%) (Figure 3b). Similar patterns of separation between Brazilian and Costa Rican reef profiles were also evident in the PC2 vs. PC3 and PC1 vs. PC3 plots, reinforcing the trend of distinction between the sites based on benthic cover attributes. According to the DFA results, the attributes foliose algae (foa), hard corals (hc), articulated algae (aa), dead coral (deco), gravel (gra), filamentous algae (turf) and sand (san) were the variables that showed discriminatory power, in decreasing order, in determining reef cover for the reefs off the coast of Brazil and the Caribbean of Costa Rica.

## 4 DISCUSSION

The results of this study suggest a potential association between lionfish presence and patterns in cryptobenthic fish assemblages, based on two main observations. First, cryptobenthic fish abundance was lower at sites where lionfish were more abundant, such as within Cahuita National Park. Second, comparisons between reef sites in Brazil (absence of lionfish at the time of sampling) and Costa Rica (established lionfish populations) revealed consistent differences in cryptobenthic fish abundance and composition. Although substrate characteristics differed between regions, reefs in both areas exhibited structural features associated with suitable habitat for cryptobenthic fishes, including high rugosity, availability of refuges and diverse benthic growth forms. These features are known to support cryptobenthic assemblages by providing microhabitats for shelter and persistence (Horn et al., 1999). Despite these broadly suitable habitat conditions, Brazilian reefs exhibited higher abundance and richness than those in Costa Rica, suggesting that factors beyond habitat structure may contribute to the observed patterns.

Cryptobenthic fishes rely on small‐scale substrate features such as burrows and concavities for shelter from predators (Beldade et al., 2006; Harborne et al., 2012; Horn et al., 1999). Here, reef composition differed between regions in the multivariate analysis (PCA), with Caribbean reefs showing a greater contribution of hard corals and articulated algae, whereas southwestern Atlantic reefs were dominated by macroalgae and sponges in some places (Zilberg et al., 2016). Despite these compositional differences, the DFA indicated that the observed separation was driven primarily by hard substrate, living cover and substrate height, rather than by rugosity or growth‐form diversity. Moreover, rugosity, refuge availability and growth‐form diversity remained consistently high across sites, suggesting that all reefs provided structurally complex habitat capable of supporting cryptobenthic fishes. Consequently, the lower abundance of cryptobenthic fishes observed within Costa Rica, particularly inside CNP relative to adjacent unprotected reefs, is unlikely to be explained solely by substrate structure and may instead reflect the influence of biotic interactions, including predation pressure, with lionfish presence representing one plausible contributing factor. While consistent with the known feeding ecology of lionfish (Morris Jr, 2012; Sandel et al., 2015), this association should be interpreted cautiously, as its relative importance cannot be isolated from other ecological drivers within the scope of this study, such as variation in native predator assemblages and local environmental conditions (Horn et al., 1999).

Analysis of the diet of more than 300 lionfish in the Costa Rican Caribbean showed that their prey includes a wide range of reef fishes, with damselfishes (Pomacentridae), surgeonfishes (Acanthuridae) and cryptobenthic fishes (e.g., Blenniidae) among the most frequently recorded prey groups (families) (Sandel et al., 2015). At a broader scale, analyses of approximately 8000 lionfish stomachs from multiple Caribbean locations (e.g., Belize, Bahamas, Bermuda, Costa Rica, Yucatan Peninsula) indicate that cryptobenthic fishes are consistently among the most consumed prey taxa, although this does not imply selective preference (Morris Jr, 2012; Peake et al., 2018; Sandel et al., 2015). This pattern is consistent with the lower occurrence of this assemblage observed in the present study, although this relationship should be interpreted cautiously. Despite evolutionary adaptations such as camouflage and the use of crevices and burrows (Brandl et al., 2018; Horn et al., 1999), these strategies may not fully prevent predation by lionfish. In addition, behavioural traits of lionfish, including nocturnal feeding (García‐Rivas et al., 2018), may increase access to cryptobenthic fishes, which are typically less active at night (Horn et al., 1999).

Since the arrival of lionfish in Costa Rica, local communities and MPA authorities have implemented removal efforts, including fishing tournaments, as a strategy to control invasive populations (Toro, 2016; Ulman et al., 2022). However, no tournaments were conducted in 2017–2018, the period of this study, due to logistical constraints, adverse weather and legal restrictions associated with fully protected areas. During this time, lionfish removals continued opportunistically in unprotected sites (e.g., Isla Uvita) through artisanal fishing and recreational diving activities. Lower lionfish abundance observed outside the MPA coincided with higher abundance and richness of cryptobenthic fishes. Although this pattern is consistent with a potential influence of lionfish predation, other factors may also contribute. Fishing pressure in unprotected areas can affect multiple components of the reef fish community, including potential predators and competitors of cryptobenthic species, thereby influencing local assemblage structure (Horn et al., 1999). In addition, as discussed previously, differences in benthic composition and habitat characteristics among sites, as indicated by the PCA, may contribute to the observed variation. Yet, within the MPA, the absence of systematic removal efforts may have favoured the persistence of lionfish populations (Soares et al., 2025), whereas unprotected areas may have functioned as zones of incidental control.

The only survey conducted in the Costa Rican Caribbean prior to the lionfish invasion reported nearly 80 cryptobenthic individuals within ~480 m^2^ in Cahuita National Park, most of them belonging to the blenniid genus *Ophioblennius* (Phillips & Perez‐Cruet, 1984). In contrast, our study recorded only six individuals across ~4200 m^2^ in 2017–2018. This marked difference suggests a substantial decline in abundance over time. While multiple factors may contribute to this pattern, it is consistent with the period following lionfish establishment in the region. Given the important role of cryptobenthic fishes in reef food webs, a precautionary approach supports prioritizing lionfish control and removal both inside and outside protected areas (Morris Jr, 2013), particularly as the ecological consequences of invasion are still being understood (Soares et al., 2025). The influence of lionfish on reef communities is likely context‐dependent, shaped by factors such as habitat characteristics, species interactions, predator assemblages and environmental conditions (Morris Jr, 2013).

In Costa Rica, local fishermen and scuba dive tourism guides play a central role in lionfish management, as they feel they can lose the most by the presence of this invasive species. In addition to directed tournaments (Toro, 2016), some restaurants across the southern Caribbean include lionfish in their menu. This is a potentially win‐win solution, as fishermen and scuba dive guides help meet an increasing demand for fish consumption while keeping lionfish, and thus the reef health, under control. Such impromptu management measures could also be suited to Brazil, where official monitoring of such an extensive coastline may prove unfeasible. Yet, such an aggressive invasive species requires multiple management measures, from policy making to citizen science, in order to mitigate its effects on the local fishing economy, human health and tourism (Morris Jr, 2013). Citizen science, in particular, can contribute to sighting reports, participatory monitoring and community‐based removal efforts, thereby supporting early detection and rapid response (López‐Gómez et al., 2014; Phillips & Kotrschal, 2021; Scyphers et al., 2015).

To mitigate lionfish impacts, we propose four integrated strategies. First, we suggest the promotion of community‐based removal programs, such as periodic workshops and fishing competitions, especially in high‐endemism areas like the MPAs (Soares et al., 2025), as Fernando de Noronha. While short‐term ‘reward’ programs are less sustainable, recreational tournaments can foster lasting engagement among fishers, community members and tourists (Ulman et al., 2022). Second, developing commercial markets for lionfish is crucial; this involves promoting the consumption of its prized meat—despite handling challenges (Morris Jr, 2012)—and exploring alternative products like bio‐jewellery. Initial financial incentives can catalyse these markets but should not be the sole long‐term mechanism (Ulman et al., 2022). Third, reef monitoring should be enhanced by incorporating cryptobenthic fish using simplified, higher‐taxon‐level protocols to facilitate broader application and a more comprehensive assessment of reef health (Brandl et al., 2018). Finally, implementing locally adapted management plans is essential. These plans should prioritize MPAs, nursery grounds and touristic sites, with a particular focus on MPAs where lionfish populations are not actively controlled. In such areas, restrictions on human activity may inadvertently facilitate the establishment and persistence of lionfish populations (Kleitou et al., 2024). Without continuous removal efforts, invasive populations may increase and contribute to broader ecosystem impacts within and beyond protected boundaries (Soares et al., 2025). Therefore, management actions in MPAs should include targeted removal efforts and actively leverage citizen science programs, particularly through dive operators, where possible, to support early detection and monitoring.

In summary, the results indicate spatial variation in cryptobenthic fish abundance across the study areas, including structurally complex reefs that typically support high diversity. Within Costa Rica, lower abundance and richness of cryptobenthic fishes were observed inside the MPA, where higher lionfish densities were recorded and removal efforts were not consistently implemented, compared to adjacent unprotected reefs. The comparison with Brazil, where lionfish were absent at the time of sampling, further highlights differences in cryptobenthic fish assemblages across contexts and the importance of implementing control measures early in the invasion and establishment process. Given the ecological role of cryptobenthic fishes in energy transfer and trophic dynamics, changes in their abundance may have broader implications for reef functioning. Accordingly, proactive lionfish management, particularly in regions of high endemism, should be prioritized through participatory strategies integrating local communities, public agencies and scientific monitoring.

## AUTHOR CONTRIBUTIONS

LFM and TAS collected the data; JNC and MST provided logistical support; LFM and PFML outlined the study; APS and CERDA performed statistical analyses; APS, CERDA, LFM, PFML and TAS performed data interpretation and analysis; all authors wrote and revised the manuscript.

## FUNDING INFORMATION

PFML thanks CNPq for a productivity grant (302365/2022‐2) and the Romanian Ministry of Research, Innovation and Digitalization, Project No. 760054‐JUST4MPA, within the PNRR‐III‐C9‐2022‐I8. LFM and TAS thank CNPq for INCT‐Ambitropic (INCTAmbTropic CNPq 565054/2010‐4 and FAPESB 8936/201). This study was financed in part by the Coordenação de Aperfeiçoamento de Pessoal de Nível Superior—Brasil (CAPES) —Finance Code 001.

## CONFLICT OF INTEREST STATEMENT

The authors state no conflict of interest.

## Supporting information


**Annex S1.** Number of transects conducted at reef sites for the assessment of cryptobenthic ichthyofauna and benthic composition using point‐intercept transects (PIT) and habitat assessment surveys (HAS) in the Caribbean (Costa Rica) and Brazilian tropical reefs (Rio Grande do Norte). *n* = number of transects, min‐max = minimum and maximum depths.
**Annex S2.** Biodiversity descriptors calculated for the fish assemblages, including cryptobenthic species and lionfish, in the study areas located in Brazil and Costa Rica (outside and inside Cahuita National Park, CNP). Metrics include species richness, total abundance, dominance, Simpson's diversity index, Shannon's diversity index, and Pielou's evenness.
**Annex S3.** Results of the Analysis of Similarity (ANOSIM) based on the Jaccard index, using presence/absence data for cryptobenthic fish species together with lionfish records, comparing the study areas in Brazil and Costa Rica (outside and inside Cahuita National Park, CNP).
**Annex S4.** Principal component analysis cumulative variance and loadings for reef structure (HAS) and Point Intercept Transect (PIT) for the first three components. Rugosity (RUGOSI), plant and animal growth form variety (FORMS), height (HEIGHT), refuge categories (REFUGE), living cover (LCOVER), hard substrate (HSUBST), articulated algae (aa), calcareous algae (caa), foliose algae (foa), hard corals (hc), dead coral (deco), diseased coral (dico), gravel (gra), filamentous algae (turf), octocorals (oct), sand (san), seag (seagrass), spo (sponges) and zoan (zoanthids). Bold highlight modular values above 0.4 considered indicative of relevant variation.

## Data Availability

The data that support the findings of this study are available on request from the corresponding author. The data are not publicly available due to privacy or ethical restrictions.

## References

[jfb70636-bib-0001] Ahmadia, G. N. , Pezold, F. P. , & Smith, D. J. (2012). Cryptobenthic fish biodiversity and microhabitat use in healthy and degraded coral reefs in SE Sulawesi, Indonesia. Marine Biodiversity, 42, 433–442.

[jfb70636-bib-0002] Albins, M. A. , & Hixon, M. A. (2013). Worst case scenario: Potential long‐term effects of invasive predatory lionfish (*Pterois volitans*) on Atlantic and Caribbean coral‐reef communities. Environmental Biology of Fishes, 96, 1151–1157.

[jfb70636-bib-0003] Anderson, M. (2008). PERMANOVA+ for PRIMER: Guide to software and statistical methods. Primer‐E Limited.

[jfb70636-bib-0004] Batjakas, I. E. , Evangelopoulos, A. , Giannou, M. , Pappou, S. , Papanikola, E. , Atsikvasi, M. , Poursanidis, D. , & Gubili, C. (2023). Lionfish diet composition at three study sites in the Aegean Sea: An invasive generalist? Fishes, 8(6), 314.

[jfb70636-bib-0005] Beldade, R. , Erzini, K. , & Gonçalves, E. J. (2006). Composition and temporal dynamics of a temperate rocky. Journal of the Marine Biological Association of the United Kingdom, 86, 1221–1228.

[jfb70636-bib-0006] Beldade, R. , & Gonçalves, J. (2007). An interference visual census technique applied to cryptobenthic fish assemblages. Vie et Milieu – Life and Environment, 57(1/2), 61–65.

[jfb70636-bib-0007] Brandl, S. J. , Goatley, C. H. R. , Bellwood, D. R. , & Tornabene, L. (2018). The hidden half: Ecology and evolution of cryptobenthic fishes on coral reefs. Biological Reviews, 93, 1846–1873. 10.1111/brv.12423 29736999

[jfb70636-bib-0008] Brandl, S. J. , Rasher, D. B. , Côté, I. M. , Casey, J. M. , Darling, E. S. , Lefcheck, J. S. , & Duffy, J. E. (2019). Coral reef ecosystem functioning: Eight core processes and the role of biodiversity. Frontiers in Ecology and the Environment, 17(8), 445–454.

[jfb70636-bib-0009] Carvalho‐Filho, A. , Sazima, I. , Lima, S. M. Q. , Almeida, D. , Mendes, L. F. , Dias, R. M. , Britto, M. R. , & Gasparini, J. L. (2020). Review of the genus *Malacoctenus* (Actinopterygii: Labrisomidae) from the southwestern Atlantic, with description of two new species. Zootaxa, 4819(3), 499–520. 10.11646/zootaxa.4819.3.4 33056093

[jfb70636-bib-0010] Chagaris, D. , Patterson, W. , & Allen, M. (2020). Relative effects of multiple stressors on reef food webs in the northern Gulf of Mexico revealed via ecosystem modeling. Frontiers in Marine Science, 7, 513.

[jfb70636-bib-0011] Cleveland, W. S. (1993). Visualizing Data. Hobart Press.

[jfb70636-bib-0012] Cortés, J. (2016). The Caribbean coastal and marine ecosystems. In M. Kappelle (Ed.), Costa Rican ecosystems (pp. 591–617). University of Chicago Press.

[jfb70636-bib-0013] Cortés, J. N. , Fonseca, A. C. , Nivia‐Ruiz, J. , Nielsen‐Muñoz, V. , Samper‐Villarreal, J. , Salas, E. , Martínez, S. , & Zamora‐Trejos, P. (2010). Monitoring coral reefs, seagrasses and mangroves in Costa Rica (CARICOMP). Revista de Biología Tropical, 58, 1–22. 21302409

[jfb70636-bib-0014] Crec'Hriou, R. , Alemany, F. , Roussel, E. , Chassanite, A. , Marinaro, J. Y. , Mader, J. , Rochel, E. , & Planes, S. (2010). Fisheries replenishment of early life taxa: Potential export of fish eggs and larvae from a temperate marine protected area. Fisheries Oceanography, 19, 135–150.

[jfb70636-bib-0015] Cure, K. , McIlwain, J. L. , & Hixon, M. A. (2014). Habitat plasticity in native Pacific red lionfish *Pterois volitans* facilitates successful invasion of the Atlantic. Marine Ecology Progress Series, 506, 243–253.

[jfb70636-bib-0016] Dalben, A. , & Floeter, S. R. (2012). Cryptobenthic reef fishes: Depth distribution and correlations with habitat complexity and sea urchins. Journal of Fish Biology, 80, 852–865. 10.1111/j.1095-8649.2012.03231.x 22471804

[jfb70636-bib-0017] Depczynski, M. , & Bellwood, D. R. (2003). The role of cryptobenthic reef fishes in coral reef trophodynamics. Marine Ecology Progress Series, 256, 183–191.

[jfb70636-bib-0018] Depczynski, M. , & Bellwood, D. R. (2004). Microhabitat utilisation patterns in cryptobenthic coral reef fish communities. Marine Biology, 145, 455–463.

[jfb70636-bib-0019] Depczynski, M. , & Bellwood, D. R. (2005). Wave energy and spatial variability in community structure of small cryptic coral reef fishes. Marine Ecology Progress Series, 303, 283–293.

[jfb70636-bib-0020] Feitosa, J. L. L. , & Ferreira, B. P. (2015). Distribution and feeding patterns of juvenile parrotfish on algal‐dominated coral reefs. Marine Ecology, 36, 462–474.

[jfb70636-bib-0021] Figueiredo, M. A. O. , Horta, P. A. , Pedrini, A. G. , & Nunes, J. M. C. (2008). Benthic marine algae of the coral reefs of Brazil, a literature review. Oecologia Australis, 12, 258–269.

[jfb70636-bib-0068] Floeter, S. R. , Rocha, L. A. , Robertson, D. R. , Joyeux, J. C. , Smith‐Vaniz, W. F. , Wirtz, P. , Edwards, A. J. , Barreiros, J. P. , Ferreira, C. E. L. , Gasparini, J. L. , Brito, A. , Falcón, J. M. , Bowen, B. W. , & Bernardi, G. (2008). Atlantic reef fish biogeography and evolution. Journal of Biogeography, 35(1), 22–47.

[jfb70636-bib-0023] García‐Rivas, M. C. , Machkour‐M'Rabet, S. , Pérez‐Lachaud, G. , Schmitter‐Soto, J. J. , & Hénaut, Y. (2018). Age‐dependent strategies related to lionfish activities in the Mexican Caribbean. Environmental Biology of Fishes, 101, 563–578.

[jfb70636-bib-0024] Gotelli, N. J. , & Ellison, A. M. (2011). Princípios de estatística em ecologia (1st ed.). Artmed.

[jfb70636-bib-0025] Gratwicke, B. , & Speight, M. R. (2005). The relationship between fish species richness, abundance and habitat complexity in a range of shallow tropical marine habitats. Journal of Fish Biology, 66(3), 650–667.

[jfb70636-bib-0026] Green, K. W. J. , Zelbst, P. J. , Meacham, J. , & Bhadauria, V. S. (2012). Green supply chain management practices: Impact on performance. Supply Chain Management: An International Journal, 17(3), 290–305.

[jfb70636-bib-0027] Harborne, A. R. , Jelks, H. L. , Smith‐Vaniz, W. F. , & Rocha, L. A. (2012). Abiotic and biotic controls of cryptobenthic fish assemblages across a Caribbean seascape. Coral Reefs, 31(4), 977–990.

[jfb70636-bib-0028] Hill, J. , & Wilkinson, C. (2004). Methods for ecological monitoring of coral reefs (3rd ed.). Australian Institute of Marine Science.

[jfb70636-bib-0029] Horn, M. H. , Martin, K. L. M. , & Chotkowski, M. A. (1999). Intertidal fishes: Life in two worlds. Academic Press.

[jfb70636-bib-0030] Kleitou, P. , Rees, S. E. , Kletou, D. , Harris, H. E. , Cai, L. L. , Green, S. , Hadjioannou, L. , Savva, I. , Giovos, I. , Jimenez, C. , & Hall‐Spencer, J. M. (2024). Marine protected areas can increase the abundance of invasive lionfish (*Pterois miles*). Conservation Science and Practice, 6(6), 1–15.

[jfb70636-bib-0031] Kohler, U. , & Luniak, M. (2005). Data inspection using biplots. The Stata Journal, 5, 208–223.

[jfb70636-bib-0032] La Mesa, G. , Muccio, S. D. , & Vacchi, M. (2006). Structure of a Mediterranean cryptobenthic fish community and its relationships with habitat characteristics. Marine Biology, 149(2), 149–167.

[jfb70636-bib-0033] Laborel‐Deguen, F. , Castro, C. B. , Nunes, F. , & Pires, D. O. (2019). Recifes brasileiros: o legado de Laborel (Vol. 64). Museu Nacional, Série Livros.

[jfb70636-bib-0034] Lima, P. A. , Andrade, L. S. , Alencar, C. E. R. D. , Pereira, P. T. , Texeira, G. M. , & Fransozo, A. (2014). Two species of swimming crabs of the genus *Achelous* (crustacea, Brachyura): Environmental requirements determining the niche. Hydrobiologia, 727, 197–207.

[jfb70636-bib-0035] López‐Gómez, M. J. , Aguilar‐Perera, A. , & Perera‐Chan, L. (2014). Mayan diver‐fishers as citizen scientists: Detection and monitoring of the invasive red lionfish in the Parque Nacional Arrecife Alacranes, southern Gulf of Mexico. Biological Invasions, 16(7), 1351–1357.

[jfb70636-bib-0036] Luiz, O. J. , Santos, W. C. , Marceniuk, A. P. , Rocha, L. A. , Floeter, S. R. , Buck, C. E. , Klautau, A. G. C. M. , & Ferreira, C. E. L. (2021). Multiple lionfish (*Pterois* spp.) new occurrences along the Brazilian coast confirm the invasion pathway into the southwestern Atlantic. Biological Invasions, 23(10), 3013–3019.

[jfb70636-bib-0037] Maggioni, R. , Rocha, R. S. , Viana, J. T. , Giarrizzo, T. , Rabelo, E. F. , Ferreira, C. E. , Sampaio, C. L. S. , Pereira, P. H. C. , Rocha, L. A. , Tavares, T. C. L. , & Soares, M. O. (2023). Genetic diversity patterns of lionfish in the southwestern Atlantic Ocean reveal a rapidly expanding stepping‐stone bioinvasion process. Scientific Reports, 13(1), 13469. 10.1038/s41598-023-40407-y PMC10439126 37596337

[jfb70636-bib-0038] Magurran, A. E. (2004). Measuring biological diversity. Blackwell Publishing.

[jfb70636-bib-0039] Manly, B. F. (2018). Randomization, bootstrap and Monte Carlo methods in biology. Chapman and Hall/CRC.

[jfb70636-bib-0041] Mendes, L. F. (2006). História natural dos amborés e peixes‐macaco (Actinopterygii, Blennioidei, Gobioidei) do Parque Nacional Marinho do Arquipélago de Fernando de Noronha, sob um enfoque comportamental. Revista Brasileira de Zoologia, 23(3), 817–823.

[jfb70636-bib-0042] Mendes, L. F. (2007). *Ophioblennius trinitatis* (Pisces: Blenniidae) from the oceanic archipelagos of São Pedro e São Paulo, Fernando de Noronha and Atol das Rocas. Brazilian Journal of Oceanography, 55(1), 63–65.

[jfb70636-bib-0043] Mendes, L. F. , & Grimaldi, G. G. (2020). A biodiversidade e as formações recifais. In L. M. Rocha & L. E. C. Bonilha (Eds.), Proposta de Criação de Área Protegida Costeira‐Marinha no Rio Grande do Norte. Oceânica.

[jfb70636-bib-0044] Morris, J. A., Jr. (2012). Invasive lionfish: A guide to control and management. Gulf and Caribbean Fisheries Institute Special Publication Series Number 1.

[jfb70636-bib-0045] Morris, J. A., Jr. (2013). La invasión del pez león: passado, presente e futuro. In J. A. Morris, Jr. (Ed.), El pez león invasor: guía para su control y manejo. Gulf and Caribbean Fisheries Institute, Special Publication Series Number 2.

[jfb70636-bib-0046] Neves, J. M. M. , Lima, S. M. Q. , Mendes, L. F. , Torres, R. A. , Pereira, R. J. , & Mott, T. (2016). Population structure of the rockpool blenny *Entomacrodus vomerinus* shows source‐sink dynamics among ecoregions in the tropical southwestern Atlantic. PLoS One, 11(6), e0157472. 10.1371/journal.pone.0157472 PMC4910989 27309356

[jfb70636-bib-0047] Patzner, R. A. (1999). Habitat utilization and depth distribution of small cryptobenthic fishes (Blenniidae, Gobiesocidae, Gobiidae, Tripterygiidae) in Ibiza (western Mediterranean Sea). Environmental Biology of Fishes, 55(3), 207–214.

[jfb70636-bib-0048] Peake, J. , Bogdanoff, A. K. , Layman, C. A. , Castillo, B. , Reale‐Munroe, K. , Chapman, J. , Dahl, K. , Patterson, W. F., III , Eddy, C. , Ellis, R. D. , Faletti, M. , Higgs, N. , Johnston, M. A. , Muñoz, R. C. , Sandel, V. , Villasenor‐Derbez, J. C. , & Morris, J. A. (2018). Feeding ecology of invasive lionfish (*Pterois volitans* and *Pterois miles*) in the temperate and tropical western Atlantic. Biological Invasions, 20(10), 2567–2597.

[jfb70636-bib-0049] Pereira, R. , Teixeira, G. , Bertini, G. , Lima, P. , Alencar, C. , & Fransozo, V. (2017). Environmental factors influencing the distribution of three species within the genus *Persephona* leach, 1817 (crustacea, Decapoda, Leucosiidae) in two regions on the northern coast of São Paulo state, Brazil. Latin American Journal of Aquatic Research, 42(2), 307–321.

[jfb70636-bib-0050] Phillips, E. W. , & Kotrschal, A. (2021). Where are they now? Tracking the Mediterranean lionfish invasion via local dive centers. Journal of Environmental Management, 298, 113354. 10.1016/j.jenvman.2021.113354 34358939

[jfb70636-bib-0051] Phillips, P. C. , & Perez‐Cruet, M. J. (1984). A comparative survey of reef fishes in Caribbean and Pacific Costa Rica. Revista de Biología Tropical, 32(1), 95–102.

[jfb70636-bib-0053] Pinheiro, H. T. , Rocha, L. A. , Macieira, R. M. , Carvalho‐Filho, A. , Anderson, A. B. , Bender, M. G. , di Dario, F. , Ferreira, C. E. L. , Figueiredo‐Filho, J. , Francini‐Filho, R. , Gasparini, J. L. , Joyeux, J. C. , Luiz, O. J. , Mincarone, M. M. , Moura, R. L. , Nunes, J. A. C. C. , Quimbayo, J. P. , Rosa, R. S. , Sampaio, C. L. S. , … Floeter, S. R. (2018). South‐western Atlantic reef fishes: Zoogeographical patterns and ecological drivers reveal a secondary biodiversity centre in the Atlantic Ocean. Diversity and Distributions, 24(7), 951–965.

[jfb70636-bib-0054] R Core Team . (2021). R: A language and environment for statistical computing. Vienna, Austria: R Foundation for Statistical Computing. https://www.R-project.org/

[jfb70636-bib-0055] Rakocinski, C. F. , Lyczkowski‐Shultz, J. , & Richardson, S. L. (1996). Ichthyoplankton assemblage structure in Mississippi sound as revealed by canonical correspondence analysis. Estuarine, Coastal and Shelf Science, 43, 237–257.

[jfb70636-bib-0056] Rangel, C. A. , & Mendes, L. F. (2009). Review of blenniid fishes from Fernando de Noronha archipelago, Brazil, with description of a new species of Scartella (Teleostei: Blenniidae). Zootaxa, 2006, 51–61.

[jfb70636-bib-0057] Reef Check . (1996). Manual de instruções de pesquisas do Reef Check Brasil. *Programa de Monitoramento dos Recifes Brasileiros (Reef Check)* Ministério do Meio Ambiente.

[jfb70636-bib-0058] Sampaio, C. L. S. , Nunes, J. A. C. C. , & Mendes, L. F. (2004). *Acyrtus pauciradiatus*, a new species of clingfish (Teleostei: Gobiesocidae) from Fernando de Noronha archipelago, Pernambuco state, Northeastern Brazil. Neotropical Ichthyology, 2(4), 205–208.

[jfb70636-bib-0059] Sandel, V. , Martínez‐Fernández, D. , Wangpraseurt, D. , & Sierra, L. (2015). Ecology and management of the invasive lionfish *Pterois volitans/miles* complex (Perciformes: Scorpaenidae) in southern Costa Rica. Revista de Biología Tropical, 63(1), 213–221. 10.15517/rbt.v63i1.14749 26299126

[jfb70636-bib-0060] Scyphers, S. B. , Powers, S. P. , Akins, J. L. , Drymon, J. M. , Martin, C. M. , Schobernd, Z. H. , Schofield, P. J. , Shipp, R. L. , & Switzer, T. S. (2015). The role of citizens in detecting and responding to a rapid marine invasion. Conservation Letters, 8(4), 242–250.

[jfb70636-bib-0061] Soares, M. O. , Pereira, P. H. C. , Feitosa, C. V. , Maggioni, R. , Rocha, R. S. , Bezerra, L. E. A. , Duarte, O. S. , Paiva, S. V. , Noleto‐Filho, E. , Silva, M. Q. M. , Csapo‐Thomaz, M. , Garcia, T. M. , Arruda‐Júnior, J. P. V. , Cottens, K. F. , Vinicius, B. , Araújo, R. , Eirado, C. B. D. , Santos, L. P. S. , Guimarães, T. C. S. , … Giarrizzo, T. (2023). Lessons from the invasion front: Integration of research and management of the lionfish invasion in Brazil. Journal of Environmental Management, 340, 1–11. 10.1016/j.jenvman.2023.117954 37119623

[jfb70636-bib-0062] Soares, M. O. , Pereira, P. H. C. , Rabelo, E. F. , Sampaio, C. L. S. , Xavier, A. L. , Mendes, L. F. , Nunes, J. , Bleuel, J. , Reis‐Filho, J. A. , & Giarrizzo, T. (2025). Invasive lionfish spread through southwestern Atlantic marine protected areas. Marine Environmental Research, 208, 1–11. 10.1016/j.marenvres.2025.107099 40154143

[jfb70636-bib-0063] Timm, N. H. (2002). Applied multivariate analysis. Springer.

[jfb70636-bib-0064] Toro, M. S. (2016). Traditional fishing folk's knowledge on Costa Rica's southern Caribbean coast: A critical component in a formula for lionfish reduction. In N. Faraclas , R. Severing , C. Weijer , L. Echteld , W. Rutgers , & R. Dupey (Eds.), Celebrating multiple identities: Opting out of neocolonial monolingualism, monoculturalism and mono‐identification in the greater Caribbean (pp. 7–22). University of Curaçao and Fundashon pa Planifikashon di Idioma.

[jfb70636-bib-0065] Ulman, A. , Ali, F. Z. , Harris, H. E. , Adel, M. , Mabruk, S. A. A. A. , Bariche, M. , Candelmo, A. C. , Chapman, J. K. , Çiçek, B. A. , Clements, K. R. , Fogg, A. Q. , Frank, S. , Gittings, S. R. , Green, S. J. , Hall‐Spencer, J. M. , Hart, J. , Huber, S. , Karp, P. E. , Kyne, F. C. , … Yildiz, T. (2022). Lessons from the western Atlantic lionfish invasion to inform management in the Mediterranean. Frontiers in Marine Science, 9(865162), 1–12.

[jfb70636-bib-0066] Zilberg, C. , et al. (2016). Conhecendo os recifes brasileiros: rede de pesquisas Coral Vivo. Série Livros, Museu Nacional, UFRJ.

[jfb70636-bib-0067] Zuur, A. F. , Leno, E. N. , & Elphick, C. S. (2010). A protocol for data exploration to avoid common statistical problems. Methods in Ecology and Evolution, 1, 3–14.

