# Supplementary material for: Cryptobenthic reef fishes may not be safe from invasive lionfish (Pterois spp. Oken, 1817)
Source: J Fish Biol. 2026 Sep 1;109(4):2488–97. doi: 10.1111/jfb.70636 (PMC13675501; doi:10.1111/jfb.70636)
Supplement: Supplementary file 1 [file JFB-109-2488-s001.docx]

**Supplementary Materials**

**Cryptobenthic reef fishes may not be safe from invasive lionfish (*Pterois* spp*.* Oken, 1817)**

Annex S1 - Number of transects conducted at reef sites for the assessment of cryptobenthic ichthyofauna and benthic composition using point-intercept transects (PIT) and habitat assessment surveys (HAS) in the Caribbean (Costa Rica) and Brazilian tropical reefs (Rio Grande do Norte). n = number of transects, min-max = minimum and maximum depths

| Country | Treatment | Site Name | Area Sampled | Sample Size (n) | Depth Range (m) |
| --- | --- | --- | --- | --- | --- |
| **Costa Rica (4200 m²)** | CNP | Peresozo | 600 m² | 10 | 0.8 – 1.5 |
|  | CNP | Punta Cahuita | 600 m² | 10 | 0.7 – 1.3 |
|  | CNP | Borde de la casa | 180 m² | 3 | 1.0 – 1.3 |
|  | CNP | Galeones | 420 m² | 7 | 0.8 – 3.5 |
|  | CNP | Pollito | 600 m² | 10 | 2.5 – 4.0 |
|  | CNP | Eduardo | 600 m² | 10 | 2.5 – 4.0 |
|  | Off CNP | Manzanillo | 600 m² | 10 | 0.8 – 1.5 |
|  | Off CNP | Isla Ulvita | 600 m² | 10 | 2.5 – 4.0 |
| **Brazil (3600 m²)** | — | Tabatinga | 600 m² | 10 | 0.5 – 2.0 |
|  | — | Ilha Verde | 600 m² | 10 | 0.5 – 2.0 |
|  | — | Pirambúzius | 600 m² | 10 | 0.5 – 2.0 |
|  | — | Pirangi | 600 m² | 10 | 0.5 – 3.0 |
|  | — | Rio do Fogo | 600 m² | 10 | 1.5 – 4.0 |
|  | — | Maracajaú | 600 m² | 10 | 1.5 – 4.0 |

Annex S2 - Biodiversity descriptors calculated for the fish assemblages, including cryptobenthic species and lionfish, in the study areas located in Brazil and Costa Rica (outside and inside Cahuita National Park, CNP). Metrics include species richness, total abundance, dominance, Simpson's diversity index, Shannon's diversity index, and Pielou's evenness.

|  | Brazil | off CNP | CNP |
| --- | --- | --- | --- |
| Richness | 7 | 6 | 7 |
| Abundance | 164 | 32 | 19 |
| Dominance | 0.2296 | 0.2793 | 0.2853 |
| Simpson's diversity index | 0.7704 | 0.7207 | 0.7147 |
| Shannon's diversity index | 1.6133 | 1.4660 | 1.5570 |
| Pielou's evenness | 0.8291 | 0.8182 | 0.8000 |

Annex S3 - Results of the Analysis of Similarity (ANOSIM) based on the Jaccard index, using presence/absence data for cryptobenthic fish species together with lionfish records, comparing the study areas in Brazil and Costa Rica (outside and inside Cahuita National Park, CNP).

|  | Brazil | off CNP | CNP |
| --- | --- | --- | --- |
| Brazil |  | < 0.01 | < 0.01 |
| off CNP | < 0.01 |  | 0.0954 |
| CNP | < 0.01 | 0.0954 |  |

Annex S4 – Principal component analysis cumulative variance and loadings for reef structure (HAS) and Point Intercept Transect (PIT) for the first three components. Rugosity (RUGOSI), plant and animal growth form variety (FORMS), height (HEIGHT), refuge categories (REFUGE), living cover (LCOVER), hard substrate (HSUBST), articulated algae (aa), calcareous algae (caa), foliose algae (foa), hard corals (hc), dead coral (deco), diseased coral (dico), gravel (gra), filamentous algae (turf), octocorals (oct), sand (san), seag (seagrass), spo (sponges), and zoan (zoanthids). Bold highlight modular values above 0.4 considered indicative of relevant variation.

*Reef structure (HAS*)

|  | PC1 | PC2 | PC3 |
| --- | --- | --- | --- |
| Standard deviation | 1.817 | 1.050 | 0.924 |
| Proportion of Variance | 55.05% | 18.38% | 14.24% |
| Cumulative Proportion | 55.05% | 73.43% | 87.67% |
|  |  |  |  |
| *Loadings* | PC1 | PC2 | PC3 |
| RUGOSI | **0.503** | -0.057 | 0.234 |
| FORMS | 0.384 | -0.063 | -0.618 |
| HEIGHT | **0.474** | -0.151 | 0.393 |
| REFUGE | 0.223 | **0.771** | 0.375 |
| LCOVER | 0.343 | **0.435** | -0.518 |
| HSUBST | **0.456** | **-0.431** | 0.062 |

*Point Intercept Transect (PIT)*

|  | PC1 | PC2 | PC3 |
| --- | --- | --- | --- |
| Standard deviation | 23.514 | 17.033 | 15.085 |
| Proportion of Variance | 41.72% | 21.89% | 17.17% |
| Cumulative Proportion | 41.72% | 63.62% | 80.79% |
|  |  |  |  |
| *Loadings* | PC1 | PC2 | PC3 |
| aa | 0.085 | 0.107 | -0.041 |
| caa | -0.036 | -0.031 | -0.103 |
| foa | **-0.716** | 0.188 | -0.383 |
| san | -0.191 | **-0.361** | 0.759 |
| gra | -0.128 | 0.143 | -0.038 |
| hc | **0.502** | **0.629** | 0.131 |
| dico | 0.006 | 0.010 | 0.002 |
| deco | -0.030 | -0.173 | 0.133 |
| Oct | 0.021 | 0.024 | 0.016 |
| seag | -0.004 | 0.009 | 0.003 |
| spo | 0.069 | 0.039 | 0.001 |
| Turf | **0.410** | **-0.612** | -0.478 |
| zoan | 0.011 | 0.009 | 0.006 |
